# Does Habitat Heterogeneity in a Multi-Use Landscape Influence Survival Rates and Density of a Native Mesocarnivore?

**DOI:** 10.1371/journal.pone.0100500

**Published:** 2014-06-25

**Authors:** Eric M. Gese, Craig M. Thompson

**Affiliations:** 1 United States Department of Agriculture, Wildlife Services, National Wildlife Research Center, Department of Wildland Resources, Utah State University, Logan, Utah, United States of America; 2 Department of Wildland Resources, Utah State University, Logan, Utah, United States of America; Smithsonian Conservation Biology Institute, United States of America

## Abstract

The relationships between predators, prey, and habitat have long been of interest to applied and basic ecologists. As a native Great Plains mesocarnivore of North America, swift foxes (*Vulpes velox*) depended on the historic disturbance regime to maintain open grassland habitat. With a decline in native grasslands and subsequent impacts to prairie specialists, notably the swift fox, understanding the influence of habitat on native predators is paramount to future management efforts. From 2001 to 2004, we investigated the influence of vegetation structure on swift fox population ecology (survival and density) on and around the Piñon Canyon Maneuver Site, southeastern Colorado, USA. We monitored 109 foxes on 6 study sites exposed to 3 different disturbance regimes (military training, grazing, unused). On each site we evaluated vegetation structure based on shrub density, basal coverage, vegetation height, and litter. Across all sites, annual fox survival rates ranged from 0.50 to 0.92 for adults and 0.27 to 0.78 for juveniles. Among sites, population estimates ranged from 1 to 7 foxes per 10 km transect. Fox density or survival was not related to the relative abundance of prey. A robust model estimating fox population size and incorporating both shrub density and percent basal cover as explanatory variables far outperformed all other models. Our results supported the idea that, in our region, swift foxes were shortgrass prairie specialists and also indicated a relationship between habitat quality and landscape heterogeneity. We suggest the regulation of swift fox populations may be based on habitat quality through landscape-mediated survival, and managers may effectively use disturbance regimes to create or maintain habitat for this native mesocarnivore.

## Introduction

Historically, North American grasslands and shrub-steppe systems were maintained through the interactions of frequent, low intensity disturbances such as fire, native herbivore grazing, drought, and soil disturbances [1], [2]. These interactions resulted in a mosaic of different-aged grasslands across the landscape [3], which benefited native wildlife [4], and enhanced community richness and diversity [5]. However, during the 1900s natural grassland systems in the Great Plains of North America were altered through processes such as the conversion of prairie into ranchland and cropland, fire suppression, and predator control programs [6]. The alterations interacted to create a variety of landscape changes including the conversion of native grassland to shrubland [7] and the homogenization of the landscape [5]. Concurrently, swift fox (*Vulpes velox*) populations declined and by 1950 they were believed to be absent from much of their historic range [8]. In 1978 the swift fox was declared extirpated in the Canadian prairies [9].

While the direct effects of disturbances on native species are often limited, indirect effects mediated through changes in vegetation structure are thought to have a much greater effect [2]. Since the mid-1970s, extensive research has focused on swift fox distribution and demographics [8], [10]–[14]. However, much of this has focused on the characteristics of individual populations, leaving a large gap in the understanding of landscape-level influences [14], [15]. Lately, researchers have investigated the influence of landscape variation on swift fox ecology, or compared spatial ecology and demographics across habitat types [11], [13], [14], [16]–[18]. Viewed as shortgrass specialists, swift foxes have been shown to be capable of exploiting a variety of habitats and prey [8], [19], [20].

In 1982 the United States Army purchased 1,040 km^2^ of southeastern Colorado grassland for the purpose of mechanized infantry training. Since then, livestock have been excluded from the area, and fire suppression increased. Military training activity commenced in 1985 on the site, primarily in the form of mechanized infantry [21]. Due to the scale of training maneuvers, some areas of the base were underutilized resulting in some areas being disturbance-free. Research on the response of the vegetative community to this change in ownership and land use has identified two interacting landscape trajectories: an increase in basal cover and grass height following the release from grazing and a reduction in basal cover, shrub height, and shrub density associated with military training [21], [22].

The objective of our research was to investigate the influence of vegetation structure on swift fox population ecology, principally survival rates and density, on and around the U.S. Army Piñon Canyon Maneuver Site, southeastern Colorado, USA. The abrupt shift in land ownership, the discrete boundaries of the training area, and the patterns of land use within the military parcel coalesced into a natural experiment on the influence of landscape heterogeneity and vegetation structure on swift fox ecology. While there was no true experimental control of treatments in our study, due to the temporal and spatial scale of terrestrial vertebrate research, observational studies following landscape-level changes are often the only available option. We therefore use the term ‘natural experiment’ cautiously; our research was observational yet capitalized on a well-defined change in land use practices and the resulting changes in landscape structure and swift fox demographics.

## Methods

### Ethics Statement

Fieldwork was approved and sanctioned by the United States Department of Agriculture's National Wildlife Research Center, the United States Army – Directorate of Environmental Compliance and Management, and the United States Forest Service. Permission to access land on the Piñon Canyon Maneuver Site was obtained from the United States Army, permission to access land of the Comanche National Grassland was obtained from the United States Forest Service, and permission to access private land was obtained from the landowner.

Capture and handling protocols were reviewed and approved by the Institutional Animal Care and Use Committees (IACUC) at the United States Department of Agriculture's National Wildlife Research Center (QA-930) and Utah State University (#1060). Permits to capture and handle swift foxes and small mammals were obtained from the Colorado Division of Wildlife (state license numbers 01-TR001, 02-TR001, 03-TR001, 04-TR001). Data were archived with the United States Department of Agriculture's National Wildlife Research Center (QA-930) and is available with permission from the authors.

### Study Area

**Figure 1:**
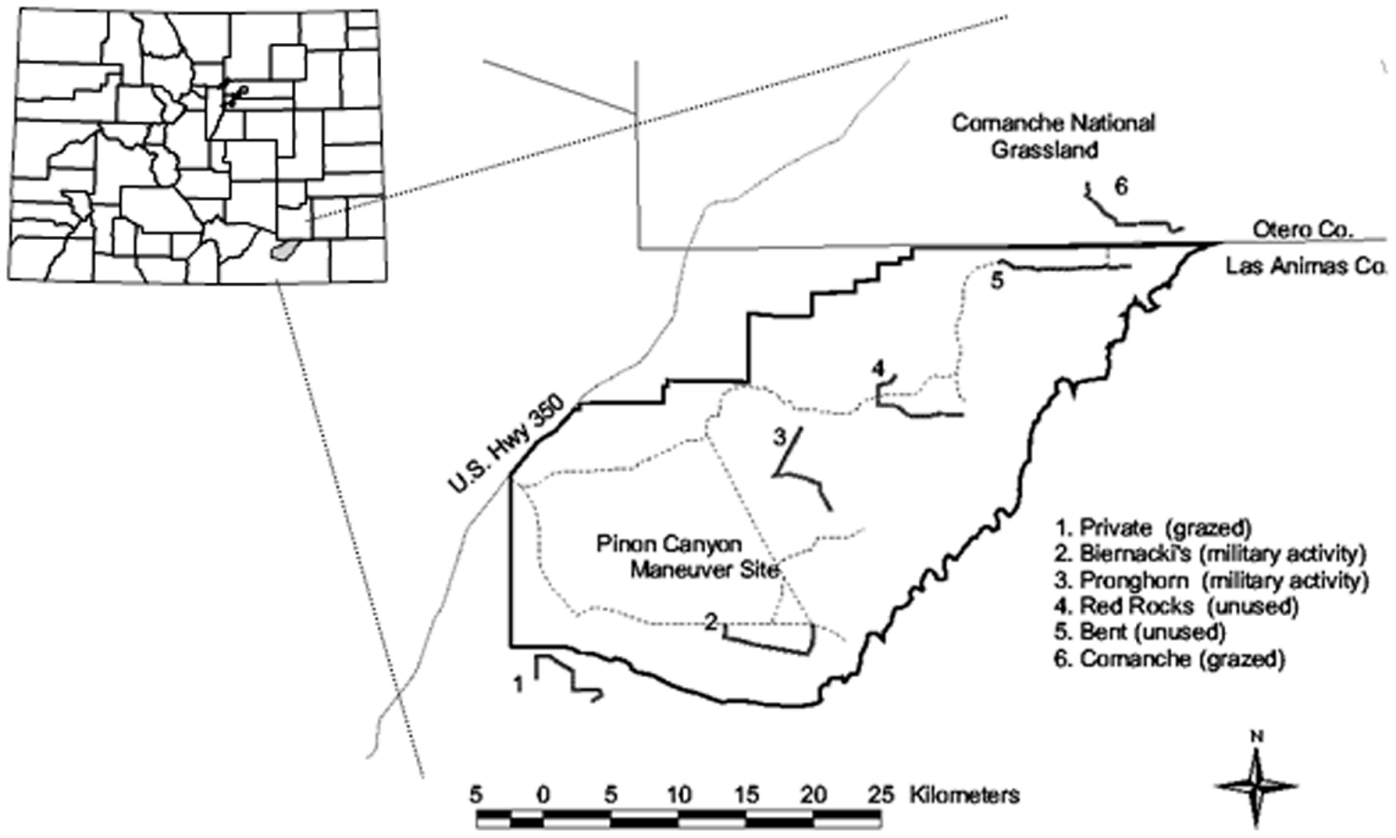
Six study sites on and around the Piñon Canyon Maneuver Site, southeastern Colorado, USA. Locations of the 6 transects are indicated, as well as the associated dominant land use.

The study area was on and around the 1,040-km^2^ Piñon Canyon Maneuver Site (PCMS) located in Las Animas County, Colorado, USA, plus areas on the United States Forest Service Comanche National Grassland, and private ranchland (Fig. 1). The region was classified as semi-arid grassland steppe, with approximately 60% categorized as shortgrass prairie dominated by blue grama (*Bouteloua gracilis*), western wheatgrass (*Pascopyrum smithii*), and galleta (*Hilaria jamesii*) [23]. Shrublands interspersed throughout the area included four-winged saltbrush (*Atriplex canescens*) and greasewood (*Sacrobatus vermiculatus*), plus prickly pear cactus (*Opuntia phaeacantha*), cholla (*Opuntia imbricata*), and yucca (*Yucca glauca*). The remaining landscape was dominated by pinyon-juniper woodlands (*Pinus edulis, Juniperus monosperma*). Elevation varied between 1,310 to 1,740 m, average temperatures ranged from 1°C in January to 23°C in July, and precipitation averaged 30 cm [21]. Monthly precipitation was highest in July with an average of 4.3 cm of rain, though the 35% of the annual precipitation that fell during the cool-season (March-May) had a proportionally greater impact on productivity [22].

### Study Design

In order to deal with the range of spatial scales used by predators and prey, we developed a hierarchical study design. We identified 6 study sites in areas subjected to 3 land use regimes: livestock grazing, mechanized military training, and unused. Unused areas were considered controls despite the fact that ‘no disturbance’ is an unnatural state for grassland ecosystems; these were sites on military property and protected from grazing, yet were not used for training purposes. Two study sites were located in each land use regime and sites were named according to landmarks or historical owners: Private (PRV), Biernacki's (BTS), Pronghorn (PRN), Red Rocks (RRK), Bent (BNT), and Comanche (COM). Each study site was centered around a 10-km trapping transect [24], [25], and the outer boundaries were defined by the home ranges of resident swift foxes [24]. Within each site, we randomly placed 50×70 m sampling grids within 1 km of the trapping transect. We used a random number generator to create a distance along the trapping transect, a direction (right or left), and a distance from the transect. This point became the northwest corner of the grid. These grids served as sampling units for both small mammal trapping and vegetation structure surveys. Each study site was considered to be spatially independent (i.e., home ranges of foxes did not overlap adjacent transects, nor did foxes travel beyond one transect during a season).

Swift fox populations on each site were evaluated based on density and survival rates [24], [25]. Each year was divided into 3 seasons based on fox behavior: breeding/gestation: 15 December – 14 April; pup-rearing: 15 April – 14 August; dispersal: 15 August – 14 December [25], [26]. We calculated both overall and seasonal estimates of population density and survival rates [24], [25]. Small mammal and vegetation surveys were also conducted seasonally at a rate of 4 grids per site per season, resulting in 12 grids sampled/site/year [27]. While we assumed differences in vegetation structure resulted primarily from differences in land use, each study site was considered an experimental unit due to the intrinsic small scale variation between them. We attempted to minimize the effect of within-site heterogeneity through replication and the distribution of sites; however additional uncontrollable and confounding factors such as disturbance intensity, cattle stocking rates, and the degree of fire suppression precluded the use of a treatment – control design.

### Swift Fox Capture and Radio-Telemetry

We captured foxes using box traps baited with chicken [28]. Traps were placed 500 m apart along each 10 km trapping transect resulting in 21 trap locations per study site. Each trap was oriented and covered with brush to provide protection from exposure. Traps were set in the late afternoon, checked early the following morning, and left closed throughout the day. Each site was trapped for 4 consecutive nights 3 times per year. For recollaring or targeting animals, a trap-enclosure system was used at den sites [29]. We used subsequent home range analyses to identify gaps between resident swift fox territories, and we trapped these gaps to assure full population monitoring. Captured foxes were handled without anesthesia, weighed, sexed, and aged through tooth wear (adult, juvenile). Foxes were considered juvenile until the pup-rearing season following their birth (15 April). Foxes were ear-tagged and radio-collared with 30–50 g radio transmitters (Advanced Telemetry Systems, Isanti, MN, USA). Attempts were made to remove transmitting radio-collars at the end of the study.

We located foxes a minimum of 3 times per week, twice during nighttime hours when animals were actively hunting and once during daylight hours to locate den sites. Locations were considered independent when separated by >4 hours [30]; more than sufficient time for a fox to cross its home range. Nocturnal locations were estimated using triangulation of 2–3 bearings within 5 minutes and separated by at least 40°. Triangulation was done using Program Locate II (Pacer, Truro, Nova Scotia); telemetry error on the study area was ±8° as determined from reference transmitters [24], [25]. Diurnal locations were collected visually by approaching the animal until either a den could be identified, or the animal was seen. Mortality sensors within transmitters indicated when a collar had been stationary for 4–6 hours. When a mortality signal was detected, the transmitter was recovered immediately and the location was recorded. Efforts to determine the cause of death included searching the area for tracks and other sign, as well as necropsy of any remains [31].

### Vegetation Structure

Vegetation structure has been defined as the “height, density, biomass, and dispersion of herbaceous and woody vegetation” [32]. For each of the 6 study sites, we evaluated vegetation structure based on the 50×70 m sampling grids randomly located within 1 km of the trapping transect [27]. Four grids were sampled each season, and new grids were selected each subsequent season. Each grid consisted of seven 50-m line-transects oriented north-south and spaced 10 m apart. On each line transect, vegetation type and height was evaluated by dropping a measuring pin every 1 m and recording the type and height of the tallest vegetation encountered [33]. For each grid, point measurements were combined into estimates of percent basal cover, percent bare ground, percent litter (dead material), and mean shrub and grass height. Shrub density was calculated by counting all woody plants >20 cm high within the grid. Grid estimates were combined into seasonal and annual averages for each study site. Standard deviations of grid estimates for each study site were used to represent the homogeneity of vegetation characteristics across each study site.

### Prey Base

Following vegetation sampling, we placed 35 Sherman live traps with 10 m spacing throughout the 50×70 m sampling grid. Traps were baited with equine sweet feed (corn, oats, molasses). Trapping grids were run for 4 consecutive nights; checked and closed each morning and reset each afternoon. Captured rodents were marked with Sharpie pens on the tail and abdomen allowing for identification of recaptures over the 4-day trapping period [2], [34], [35]. Relative abundance for each species was estimated based on the number of individuals captured. We calculated community richness as the number of species captures and we estimated community diversity using the Shannon-Weaver index [36].

### Data Analysis

We estimated average seasonal survival rates for juvenile and adult swift foxes, as well as an overall survival rate for each of the 6 sites using the known fate model in Program MARK [37]. The model was age-structured, allowing juveniles to graduate into the adult cohort after surviving through April of the year following their birth. Individuals not located during a season were censored for that season.

We estimated the number of foxes in each site using the robust model in Program MARK [37] and Huggin's estimator. Seasonal survival estimates for each site were taken from the known fate model. Estimates of the number of foxes were converted into density estimates by calculating the ‘effective trapping area’ associated with each transect [24], [25]. The radius of the average seasonal 95% kernel home range for foxes associated with each transect was used to buffer the transect in ArcView GIS. The resulting polygon was considered the ‘effective trapping area’ for that transect. The estimated number of foxes per transect could then be converted into a density estimate for each site. Density estimates were consolidated into seasonal averages as well as an overall estimate for each site. Chi-square analysis was used to test for differences in capture rates among sites.

We used Pearson correlation coefficients to identify vegetation variables for final analysis. We selected variables based on their independence and ability to discriminate among study sites. We evaluated seasonal differences in vegetation structure among sites using the GLM procedure carried out in SAS v9.2 and separated sites into statistically significant groupings. Due to the large number of models generated, Tukey's studentized range was used to control for the experiment-wise error rate.

We compared seasonal swift fox population parameters to seasonal vegetation variables using both univariate and multivariate techniques. We used linear regression to compare seasonal fox survival and population density with seasonal vegetation variables. We also constrained the above mentioned MARK models using combinations of grass height, shrub density, percent basal cover, and percent litter in order to further evaluate the effect of vegetation structure on fox demographics. The logit link function was used to run constrained models. We used likelihood ratio tests and AIC statistics [38] to evaluate whether the inclusion of vegetation data improved the explanatory power of the original, unconstrained known fate and robust models.

## Results

Between 20 November 2001 and 27 November 2004, 116 swift foxes were captured 238 times; 109 foxes were fitted with radio-collars. Captures were not distributed equally among sites (χ^2^ = 26.6, df = 5, *P*<0.001), with 86% of all captures occurring on the grazed or military sites (Table 1); trapping effort was equal across all study sites. Fewer foxes and a greater proportion of juvenile foxes were captured in unused sites compared to military or grazed sites (Table 1). Throughout the study, 7595 locations were recorded on the 109 collared foxes. The mean number of days a fox was monitored, from radio-collaring to either death, loss of signal, or radio-collar removal, was 299 days (SD  =  284.5). A total of 55 swift foxes died during the study (38 adult, 17 juvenile). Of these deaths, 24 (44%) were suspected coyote predation, 22 (40%) were confirmed coyote predation, 3 (5%) were badger predation, 3 (5%) were vehicle collision, 2 (4%) were golden eagle predation, and 1 (2%) was bobcat predation. Many of the suspected coyote predation events were when we recovered a torn, bloody, or buried radio-collar and were unable to conduct a necropsy. Thus, suspected and confirmed predation by coyotes accounted for 84% of the swift fox deaths with predation being the main cause of death across all study sites.

**Table 1:** Number, age, and sex ratios of swift foxes captured in 6 sites in southeastern Colorado, USA, 2001–2004.

Dominant land use	Site	# animals captured	Males: Females	Proportion adults
Grazed	PRV	32	18/14	0.44
	COMM	17	7/10	0.41
Military	BTS	23	9/14	0.57
	PRN	28	14/14	0.36
Unused	RRK	9	3/6	0.22
	BNT	7	5/2	0.29

### Prey Base and Swift Fox Survival and Density

Small mammal communities were sampled on 185 trapping grids. Northern grasshopper mice (*Onychomys leucogaster*), Ord's kangaroo rat (*Dipodomys ordii*), silky pocket mice (*Perognathus flavus*), western harvest mice (*Reithrodontomys megalotis*), white-footed mice (*Peromyscus leucopus*), southern plains woodrat (*Neotoma micropus*), thirteen-lined ground squirrels (*Spermophilus tridecemlineatus*), deer mice (*Peromyscus maniculatus*), and spotted ground squirrels (*Spermophilus spilosoma*) accounted for >99% of all captures. Three species, Northern grasshopper mice, deer mice, and Ord's kangaroo rat, accounted for 76% of all captures. Only one small mammal parameter, relative abundance of Northern grasshopper mice, differed significantly between sites (*F* = 2.62, df = 5, 179, *P* = 0.03) (Table 2) and it was unrelated to either fox density (*F* = 0.002, df = 5, 179, *P* = 0.96), or fox survival (*F* = 1.60, df = 4, *P* = 0.29).

**Table 2:** Mean (± SD) vegetation structure and small mammal population parameters for 6 study sites in southeastern Colorado, USA, 2001–2004.

	Grazed		Military		Unused	
	PRV	COM	BTS	PRN	RRK	BNT
% basal cover	38.0±19.9	45.1±18.9	40.3±17.2	35.3±12.8	43.8±18.1	43.7±21.7
% bare ground	42.6±19.5^a^	26.1±13.9^b^	40.0±12.9^a^	37.0±11.7^a^	29.5±11.4^b^	30.6±13.8^b^
% litter	18.0±8.9^a^	24.9±14.1^b^	18.2±13.5^a^	26.4±13.8^b^	23.4±13.2^b^	16.9±9.3^a^
Mean veg. ht	7.7±5.0^a^	21.5±43.4^b^	10.2±5.1^a^	9.6±4.4^a^	16.9±24.3^b^	16.3±8.8^b^
Mean grass ht	6.7±3.9^a^	10.6±3.1^b^	9.5±5.1^b^	8.9±4.3^b^	9.4±3.0^b^	12.8±4.9^c^
Mean shrub ht	12.9±20.9^a^	59.1±62.8^b^	18.8±17.2^a^	17.1±15.4^a^	53.4±79.1^b^	42.4±65.1^b^
Shrubs/100 m^2^	0.9±2.2^a^	2.7±2.0^b^	0.7±1.0^a^	0.3±0.3^c^	0.7±0.8^a^	1.2±0.9^a^
Total Captures	1.0±1.3	2.6±3.5	2.0±3.0	2.2±2.8	3.4±8.2	4.2±6.0
NGM	0.2±0.4^a^	0.1±0.4^a^	1.0±1.7^b^	0.7±1.1^b^	0.5±1.1^a,b^	0.6±1.3^b^
DM	0.1±0.4	1.0±1.9	0.3±0.6	0.1±0.3	1.9±6.0	1.3±2.5
OKR	0.1±0.4	0.9±1.4	0.5±1.2	1.0±2.4	0.3±0.7	1.1±2.5
Richness	0.8±1.1	1.2±1.6	0.9±1.2	1.1±1.0	1.0±1.3	1.6±1.9
Diversity	0.2±0.4	0.3±0.5	0.2±0.4	0.2±0.4	0.2±0.3	0.4±0.6

Values are averages of 36 sampling grids/site. Heights are given in centimeters. Letters refer to statistically significant (*P*≤0.05) groupings for each parameter. NGM: Northern grasshopper mouse; DM: deer mouse; OKR: Ord's kangaroo rat.

### Vegetation Structure and Swift Fox Survival and Density

Between 2001 and 2004, 185 vegetation grids were sampled across the 6 study sites. Mean vegetation height ranged from 7.7 cm in the PRV site to 21.5 cm in the COM site and shrub density ranged from 0.03 (PRN) to 2.7 shrubs/100 m^2^ (COM) (Table 2). Only one vegetation parameter, percent basal cover, was not significantly different among sites (*F* = 1.38, df = 5, 179, *P* = 0.23). The remaining 6 vegetation parameters evaluated differed significantly among sites (*P*<0.01 in all cases). Groupings varied and did not correspond to the dominant land use (Table 2).

**Figure 2:**
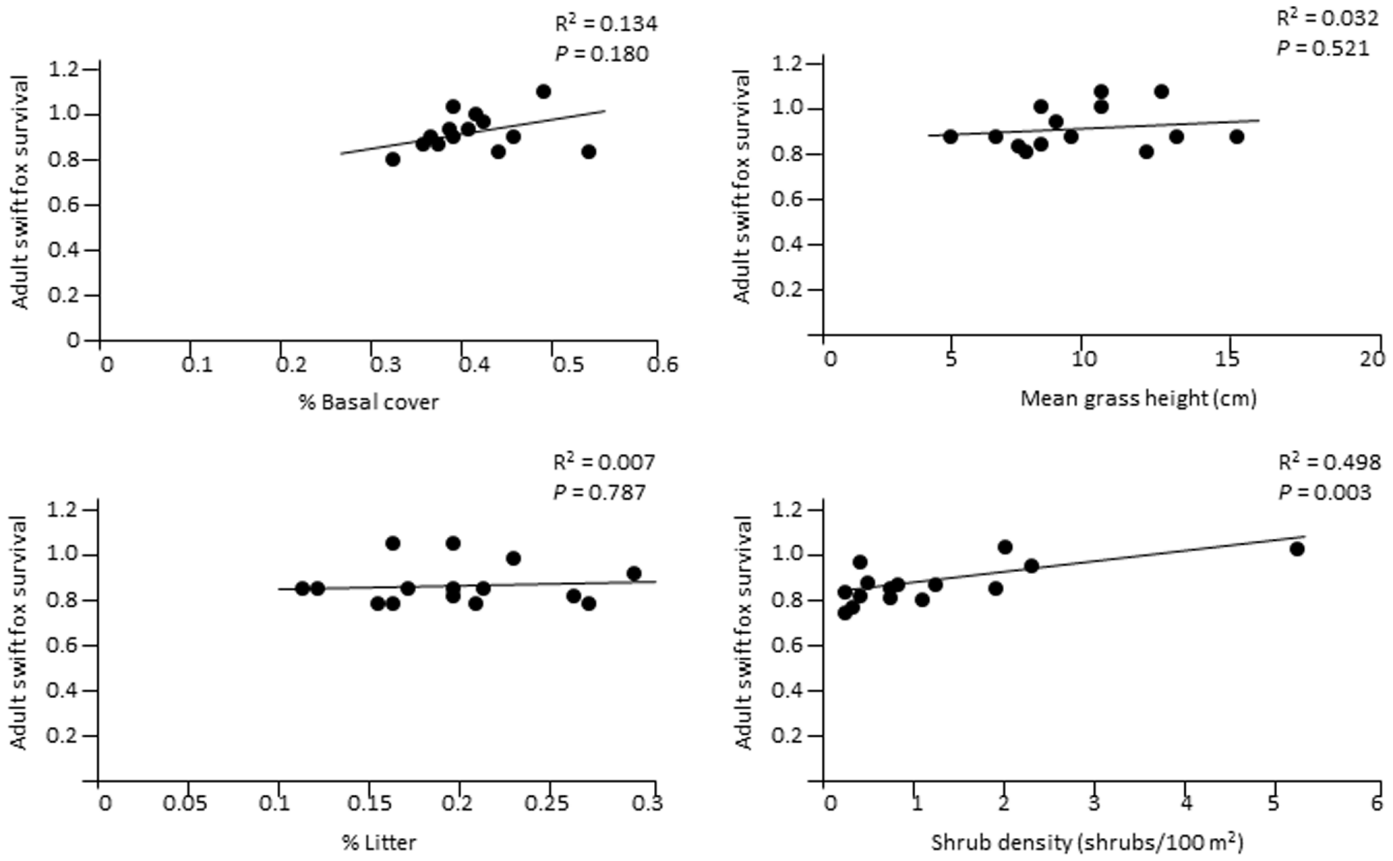
Linear regressions showing the relationships between swift fox survival rates and vegetation structure, southeastern Colorado, USA, 2001–2004.

**Figure 3:**
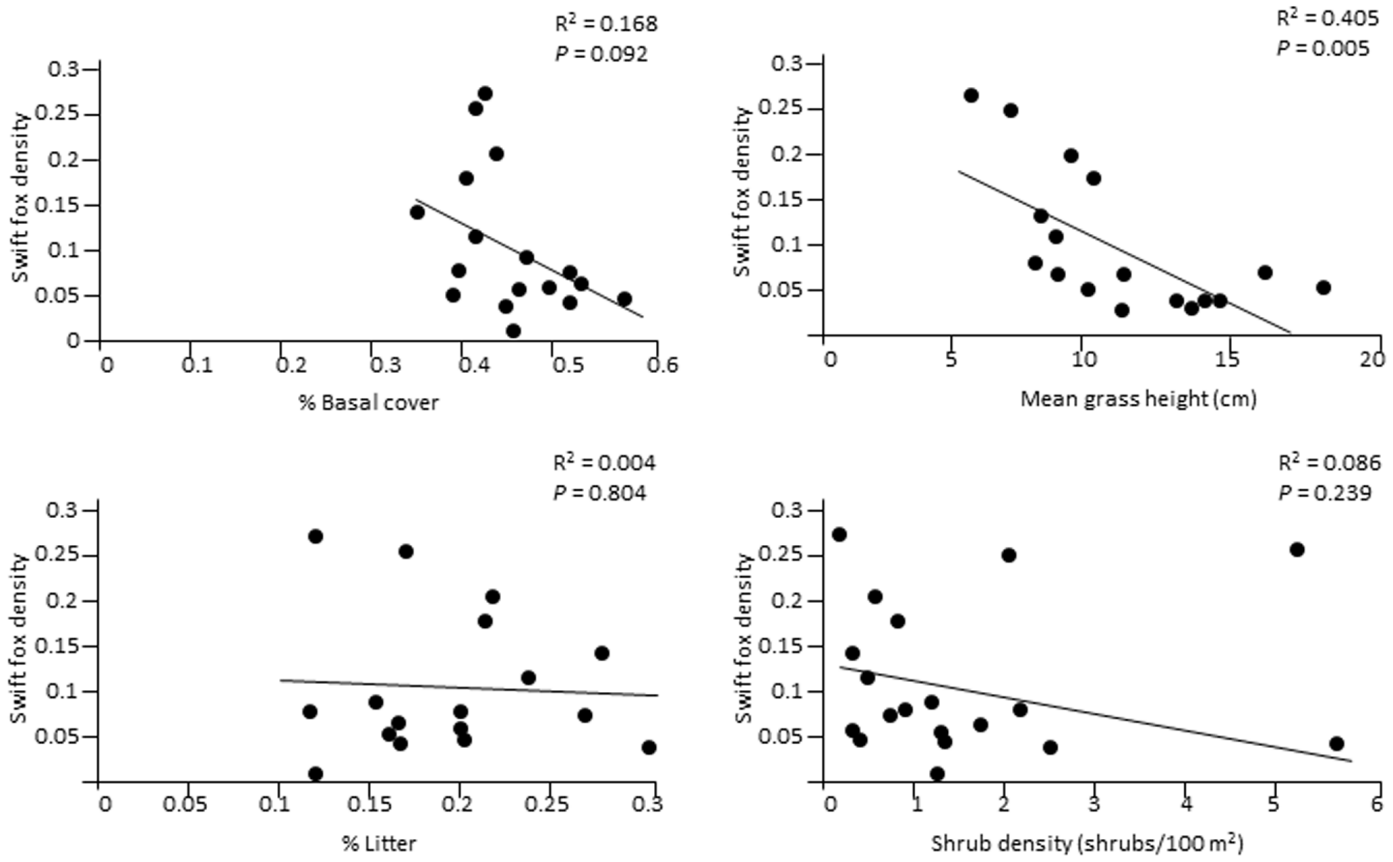
Linear regressions showing the relationships between swift fox population density and vegetation structure, southeastern Colorado, USA, 2001–2004.

Swift fox survival estimates did not differ significantly between seasons (*F* = 0.01, *P* = 0.99), by year (*F* = 0.98, *P* = 0.386), by age (*F* = 0.02, *P* = 0.891), or by site (*F* = 0.57, *P* = 0.721) (Table 3). Only one vegetation variable, shrub density, was significantly related to swift fox survival (Fig. 2). However, this relationship depended on a single outlying point. When this point was removed from the analysis, the R^2^ value dropped to 0.004 and the associated *P* value rose to 0.796. Fox population density was negatively related to all 4 vegetation variables, but only the fox density - mean grass height relationship was both statistically significant and significantly different from zero (Fig. 3).

**Table 3:** Estimates of survival rates (± SE) for adult and juvenile swift foxes on 6 study sites located in southeastern Colorado, USA, 2001–2004.

Dominant land use	Site	Age class (n)	Seasonal survival rate			Annual survival rate
			*Dispersal*	*Breeding*	*Pup rearing*	
Grazed	PRV	Adult (15)	0.81 (0.09)	0.81 (0.09)	0.83 (0.08)	0.54
		Juvenile (12)	0.89 (0.10)	0.88 (0.12)		0.65
	COM	Adult (6)	1.0 (0.0)	0.92 (0.08)	1.0 (0.0)	0.92
		Juvenile (5)	1.0 (0.0)	0.50 (0.25)		0.50
Military	BTS	Adult (12)	0.82 (0.08)	0.84 (0.08)	0.78 (0.10)	0.54
		Juvenile (3)	1.0 (0.0)	1.0 (0.0)		0.78
	PRN	Adult (11)	0.79 (0.09)	0.73 (0.11)	0.94 (0.06)	0.54
		Juvenile (11)	0.91 (0.10)	0.60 (0.15)		0.51
Unused	RRK	Adult (2)	0.75 (0.22)	0.80 (0.18)	0.83 (0.15)	0.50
		Juvenile (7)	0.33 (0.27)	1.0 (0.0)		0.27
	BNT	Adult (1)	–	–	–	–
		Juvenile (5)	–	0.75 (0.22)		–

Sample size indicates the age at capture. Juveniles surviving into April of their second year graduated into the adult cohort for analysis.

Constraining the known fate survival model, using data on shrub density, consistently improved model performance (Table 4). However, only two single-variable models, the interaction of shrub density and grass height (χ^2^ = 4.38, *P* = 0.036) and shrub density alone (χ^2^ = 4.19, *P* = 0.041), showed statistically significant improvement over the null, age-structured model based on likelihood ratio tests. These two models were roughly equivalent with ΔAICc values differing by only 0.19, and their combined AICc weight equaled 0.328. Only one additional known fate model, constrained by the interaction of shrub density and percent basal cover, had a ΔAICc value <2. No models incorporating the standard deviation of vegetation variables outperformed the null model.

**Table 4:** Results from age-structured known fate survival models, constrained by vegetation characteristics.

Model	AICc	ΔAICc	AICc weight	Model likelihood
shrub*grass	257.753	0.00	0.17208	01.0000
shrub	257.944	0.19	0.15643	0.9091
shrub*basal	258.620	0.87	0.11156	0.6483
shrub + shrub*grass	259.835	2.08	0.06076	0.3531
shrub + basal	259.924	2.17	0.05811	0.3377
shrub + litter	259.960	2.21	0.05708	0.3317
shrub + grass	259.968	2.22	0.05684	0.3303
null	260.047	2.29	0.05466	0.3176

Models shown are those that outperformed the null (age-structured) model.

Population density estimates differed by season and site (Table 5). Site most strongly influenced estimates (*F* = 5.78, df = 5, *P* = 0.004, R^2^ = 0.385). Season was marginally significant (*F* = 3.07, df = 5, *P* = 0.057); however its inclusion in the model raised the R^2^ to 0.467. One robust population density model, constrained by shrub density and percent basal cover, significantly outperformed all other models as well as the null model (χ^2^ = 39.32, *P*<0.001; Table 6). This model had an AICc weight of 0.98 and the next best performing model, constrained by shrub density alone, had a ΔAICc value of 9.68.

**Table 5:** Swift fox population density estimates (foxes/km^2^) on 6 study sites exposed to 3 land use practices in southeastern Colorado, USA, 2001–2004.

Dominant land use	Site	Seasonal density estimates			Total density estimate (SD)
		*Dispersal*	*Breeding*	*Pup-rearing*	
Grazed	PRV	0.15	0.22	0.21	0.18 (0.10)
	COM	0.03	0.03	0.06	0.04 (0.05)
Military	BTS	0.06	0.17	0.07	0.11 (0.08)
	PRN	0.04	0.11	0.09	0.09 (0.06)
Unused	RRK	0.04	0.06	0.04	0.05 (0.03)
	BNT	0.05	0.04	0.0	0.03 (0.05)

**Table 6:** Results from age-structured robust design models using Huggin's estimator to derive population size and constrained by vegetation characteristics.

Model	AICc	ΔAICc	AICc weight	Model likelihood
shrub + basal	1722.043	0.00	0.98171	1.0000
shrub	1731.718	9.68	0.00778	0.0079
grass + shrub	1733.196	11.15	0.00372	0.0038
shrub + litter	1733.855	11.81	0.00267	0.0027
shrub + sdshrub	1733.935	11.89	0.00257	0.0026
shrub + sdshrub + shrub*sdshrub	1736.055	14.01	0.00089	0.0009
grass	1736.635	14.59	0.00067	0.0007
null	1775.135	53.09	0.00000	0.0000

Models shown are those that outperformed the null (age-structured) model and resulted in an AICc weight greater than zero.

## Discussion

The swift fox survival rates we recorded were similar to those previously reported on the PCMS and elsewhere. On Piñon Canyon, estimated annual adult survival rates have ranged from 0.52 [39] to 0.88 [25]. In Wyoming, swift fox survival estimates ranged from 0.40 to 0.69 [40]. In general, our results were similar with one exception. On the Comanche site we recorded an adult survival rate of 0.92. This is one of the highest survival rates reported for swift foxes, and was based on a sample of 17 animals monitored for >3 years. On this site, population density was low, survival was high, and resident animals were larger and heavier than on other sites. While we do not have sufficient data to explain this, we speculated that effective management of the Comanche National Grassland during drought conditions resulted in the best of both worlds for swift foxes: an average grass height to allow predator detection and high shrub density to maintain prey density. While population density on the Comanche National Grassland was lower than on other sites, we believe this reflected a stable population with long-term residents and low turnover.

In contrast, estimates of juvenile survival have ranged widely. Rongstad et al. [39] estimated annual juvenile survival on PCMS at only 0.05. On the same landscape, Karki et al. [28] reported a range of survival estimates from 0.41 to 0.60. Reports have varied on whether juvenile swift foxes experience higher or lower survival than adults. Kamler et al. [41] found juvenile swift foxes had higher survival rates than adults, while Sovada et al. [16] and Schauster et al. [25] reported the opposite. Our estimates ranged from 0.27 in an ‘unused’ site to 0.78 in a site exposed to military training. The high variation in juvenile survival rates indicated fluctuating environmental conditions may play a role. For example, annual precipitation and the resulting growing season may influence juvenile survival during dispersal due to vegetation height but we did not consider a 3-year study sufficient to evaluate climate-related effects.

Density estimates on PCMS have averaged 0.22 [25] and 0.26 foxes/km^2^ [28]. These estimates were based on telemetry studies of known populations during a time when swift fox populations were believed to be at their peak. Our estimates in the same area, averaged 0.10 foxes/km^2^, were based on mark-recapture data during drought conditions. In northern Colorado, swift fox densities ranged from 0.2/km^2^ in poor habitat to 1.1/km^2^ in good habitat [42]. Our estimates ranged from 0.03 foxes/km^2^ on an ‘unused’ site to 0.18 foxes/km^2^ on a grazed site.

It is important to note that our results varied considerably among sites. While results on military-used lands were fairly consistent, swift fox population parameters on grazed lands varied widely and may have been influenced by finer scale heterogeneity. In one grazed site (COM), we recorded above average survival and below average population estimates. In the other grazed site (PRV), we recorded the highest population estimate and average survival rates. This may be related to the variation in vegetation structure among sites. In general, military sites were more homogeneous while grazed sites showed greater variation in structural measurements between vegetation grids (Table 4). ‘Grazed’ appears to be a far too simplistic category for a wildlife/landscape interaction study such as ours: individual management practices resulted in different landscape conditions and shifts between grassland and shrubland often depended on seasonal effects. During our study, drought conditions prompted the U.S. Forest Service to reduce stocking rates on the Comanche National Grassland (COM), most likely resulting in greater annual plant production compared to the PRV site where stocking rates remained constant.

At this point, extensive information exists on individual swift fox populations scattered throughout their historic range. However, there is a scarcity of information regarding the variation between these populations and what habitat factors contribute to differences in densities or demographic rates. Our results indicated a strong link between vegetation structure and swift fox population ecology, yet this link was not related to prey abundance in our study area. Population estimates were negatively related to mean grass height and adult survival was slightly positively related to shrub density. The relationship with grass height has been hinted at but not documented in previous work. For example, Kamler et al. [11] suggested the lack of swift fox activity on ungrazed Conservation Reserve Program grasslands was related to the presence of taller vegetation. They noted that even inexperienced juveniles showed an almost complete avoidance of these areas. Similarly, in our unused Bent site where mean grass height was the highest, only transient foxes were captured which were predominantly young foxes attempting to establish a home range in less habitable areas. We found that no radio-collared foxes established home ranges despite the lack of competition; all radio-collared foxes either died or left the site. The lack of resident animals in this site hindered our ability to accurately estimate survival. Our results indicated that while swift foxes were capable of exploiting a range of habitats, they showed a higher probability of population persistence in areas where disturbances kept vegetation short.

White et al. [43] documented the transition from grassland to shrubland can be accompanied by a shift from relatively rare, large bodied rodents to more abundant, small-bodied species that have fewer anti-predatory defenses. Mesocarnivores such as swift foxes may benefit from this shift due to the more abundant, vulnerable prey base, and this may explain the slight positive relationship between fox survival and shrub density. However, this benefit comes with increased risk; more shrubs generally mean reduced visibility and more jackrabbits (*Lepus californicus*), leading to increased risk of coyote predation [44]. Throughout the study, we documented dispersing swift foxes avoiding these areas of dense vegetation and assumed this indicated either an innate or learned avoidance of intraguild predation risk. At the same time, foxes living in heavily grazed areas with high shrub density and low mean grass height (COM site) were larger, heavier, and survived longer [14]. Alone, an increase in shrub density appears to carry both costs and benefits for swift foxes; increased predation risk as well as increased prey availability. The addition of increased basal cover and/or grass height, such as found in undisturbed grassland systems, appears to tip the balance and make the landscape unsuitable, presumably by increasing the risk of intraguild predation.

Recent experiments have tested the hypothesis that coyote control will result in increased swift fox survival and density with mixed results. Kamler et al. [45] reported coyote control resulted in increased swift fox survival, density, and recruitment. On the PCMS, Karki et al. [28] found coyote control resulted in increased juvenile fox survival but did not increase fox density due to compensatory dispersal, and suggested coyote control was not an effective means of increasing swift fox densities. Our results suggested an alternative, non-lethal, means of increasing swift fox population viability. Management practices oriented toward reintroducing more complex disturbance regimes such as the combination of an infrequent, intense physical disturbance and periodic prescribed burning would reduce vegetation density and grass height, and could increase the quality of habitat for swift foxes. Besides the use of prescribed burning, other disturbance regimes that might also reduce vegetation height include controlled grazing during appropriate times of the year, mechanical reduction of woody vegetation (i.e., brush management), or using crop management to reduce crop stubble. No research examining these approaches has been conducted, but warrant future consideration for managing or enhancing swift fox populations.

The relationship between grassland vegetation structure and disturbance regimes has been well established. Swift foxes evolved in grassland systems and as a result depend on grassland disturbance dynamics to maintain habitat quality in our region. Disruptions in grassland disturbance regimes have the potential to degrade swift fox habitat through long-term changes in vegetation structure [8]. A similar scenario was presented by List and Macdonald [46] for kit foxes (*Vulpes macrotis*) on Mexican grasslands, where prairie dog (*Cynomys ludovicianus*) eradication programs risk long term, indirect harm due to shrubland expansion. Our results support the evidence that swift foxes in our region are a shortgrass prairie specialist despite being capable of exploiting sub-optimal habitats [8]. We also found strong evidence of a relationship between habitat quality and landscape heterogeneity, though additional information is needed on exactly how vegetation structure influences swift fox ecology through shifts in prey base or predation pressure. We suggest the regulation of swift fox populations may be based on habitat quality through a type of landscape-mediated survival (i.e., mostly predation), and therefore managers may effectively use disturbance regimes to create or maintain habitat.
